# Effect of Aqueous Extract of Psidium Guajava Leaves on Liver Enzymes, Histological Integrity and Hematological Indices in Rats

**DOI:** 10.4021/gr2010.02.174w

**Published:** 2010-01-20

**Authors:** Friday E. Uboh, Iniobong E. Okon, Moses B. Ekong

**Affiliations:** a Biochemistry Department, Faculty of Basic Medical Sciences, University of Calabar, P.M.B.1115, Calabar, Nigeria; b Botany Department, Faculty of Sciences, University of Calabar, P.M.B.1115, Calabar, Nigeria; c Anatomy Department, Faculty of Basic Medical Sciences, University of Calabar, P.M.B.1115, Calabar, Nigeria

**Keywords:** Psidium guajava, Hepatic functions, Aminotransferases, Serum proteins, Hematopoietic

## Abstract

**Background:**

Serum alanine aminotransferase (ALT), aspartate aminotransferase(AST), alkaline phosphatase (ALP), albumin and total protein levels, as well as the tissue histological assay are known to be useful in assessing the functional integrity of the liver. Also, assessment of red and white blood cells count, hematocrit and hemoglobin concentrations is useful in determining the effect of some chemical substances on hemotopoietic system. In recent times, reports from medicinal plants research indicate that extracts from some plants are both hepatotoxic and hematotoxic, while others on the other hand are reported to be hepatoprotective and hematopoietic in action. This study considers the effects of aqueous extract of Psidium guajava (P. guajava) leaves on the histology and biochemical indices of liver function as well as hematological indices in rats.

**Methods:**

In this study, phytochemical screening of the aqueous extract of P. guajava leaves was carried out. Also, male and female rats were administered with 200 mg/kg body weight oral daily doses of aqueous extract of P. guajava leaves for a period of 30 days. At the end of the administration period, the rats were anaesthesized with chloroform vapors and dissected for the collection of blood and liver tissues which were used for the hematopoietic and liver functions investigations.

**Results:**

Preliminary phytochemical analysis of the plant leaves showed the presence of alkaloids, flavonoids, glycosides, polyphenols, reducing compounds, saponins and tannins. Liver function tests revealed that the serum ALT, AST and ALP, as well as the concentrations of total protein and albumin in male and female rats were not significantly (P > 0.05) affected by the oral administration of the extract. Histopathological study also did not show any adverse alteration in the morphological architecture of the liver tissues in both sexes of the animal model. However, red blood cell counts, hemotocrit and hemoglobin concentrations increased significantly (P < 0.05) on administration of the extract in both male and female rats. It was therefore observed that the effect of the extract on male rats was not significantly different (P > 0.05) from that obtained for the female rats.

**Conclusions:**

The results of this present study suggested that aqueous extract of Psidium guajava leaves may be hepatoprotective, and not hepatotoxic, with hematopoietic potentials in both male and female rats. These findings are therefore of clinical importance given the various reported medicinal potentials of the plant.

## Introduction

It is generally known that the consumption of a variety of local herbs and vegetables by man contributes significantly to the improvement of human health, in terms of prevention, and or cure of diseases because plants have long served as a useful and natural source of the therapeutic agents [1]. Moreover, traditional medicine is greatly relied upon especially by rural dwellers, for the treatment of various ailments: traditional doctors or healers are the dispensers of such concoctions. Guava (Psidium guajava) is a common shade tree or shrub in door-yard gardens in the tropics. The tree is easily identified by its distinctive thin, smooth, copper-colored bark that flakes off, showing a greenish layer beneath. It has been reported that the phytochemical analyses of guava leaf products, revealed the presence of more than 20 isolated compounds, including alkaloids, anthocyanins, carotenoids, essential oils, fatty acids, lectins, phenols, saponins, tannins, triterpenes, and vitamin C (80 mg per 100 g of guava) [2-6]. The main active constituent in the plant is reported to be quercetin. Spasmolytic and antidiarrheal effects are reported to be associated with its quercetin-derived, flavonoids and glycosides, which support use of this ancient leaf remedy in treating gastrointestinal disorders [7].

The decoctions made from the leaves and/or bark of Psidium guajava have been reported to be used by many tribes for diarrhea, dysentery, sore throats, vomiting, stomach upsets, vertigo, and to regulate menstrual periods throughout the tropical Amazon and India. Moreover, tender leaves are also reported to be chewed for bleeding gums and bad breath, and it is said to prevent hangovers (if chewed before drinking). According to the report, Indians throughout the Amazon gargle a leaf decoction for mouth sores, bleeding gums, or use it as a douche for vaginal discharge and to tighten and tone vaginal walls after childbirth [8, 9]. These medicinal importances of plants have been attributed to their phytochemical content. Thus phytochemcial analysis of plants is predicated by the need for drug alternatives of plant origin, made imperative by the high cost of synthetic drugs. For example, L. owariensis leaves have been reported to contain various secondary plant metabolites of medicinal value including saponins, tannins, alkaloids and flavonoids [9, 10]. These secondary plant metabolites extractable by various solvents exhibit varied biochemical and pharmacological actions in animals when ingested [11]. Within the recent decade, a good number of medicinal plants have been reported to be employed in folk medicine in the treatment of anaemia. Among these plants include Telfeira occidentalis, Combretum dolichopetalum, Allinu assalonicum, Bougainv spectabilis, Psorospermum ferbrifugum, Sorghum bicolor, Jatropha curcas, Flacourtia flavenscens, Ageratum conyzoides and Brillantasia nitens [12-18].

Anemia is one of the clinical conditions that constitute a serious health problem in many tropical countries as a result of the prevalence of different forms of parasitic infections, including malaria [19]. Anemic condition is characterized by a decrease in the level of circulating hemoglobin, less than 13 g/dl in male and 12 g/dl in females [20]. In the tropics, due to endemicity of malaria and other parasitic infections, between 10 to 20% of the population is reported to possess less than 10 g/dl of Hb in the blood [21]. The determination of hematological indices provides physiological information on a proper blood assessment. According to Okonkwo et al [22], accurate laboratory determination of blood parameters remains the only sensitive and reliable foundation for ethical and rational research, diagnosis, treatment and prevention of anemia. The major concern of the scientific communities with regard to medicinal plants and hematological studies focuses on the measures that can maintain a normal hematological state of being and reverse any negative hematological status associated with various anemic conditions. This study therefore, in part, assessed the hematopoietic potential of P. guajava leaf extract in rat model, considering the fact that different parts of the plant have been reported to be useful in the management of various diseases. On the whole, this paper aims at reporting the effects of aqueous extract of P. guajava leaves on the biochemical indices of liver function and some hematological parameters in albino Wistar rats.

## Materials and Methods

### Identification and preparation of plant Materials

Fresh leaves of P. guajava were collected in May 2009 from local garden at the University of Calabar, Calabar, Nigeria. The sample of the plant specimen was identified and authenticated by a Botanist from the botanical garden, and the Voucher specimen was deposited in the herbarium of the same University. The leaves were sorted to eliminate any dead matter and other unwanted particles. The leaves were air-dried for 2 weeks and then ground into fine powder using an electric dry mill (Moulinex). A total of 200g of the ground powder was soaked in 1 L of distilled water for 48 hours at room temperature. The mixture was filtered into 500 ml conical flask with Watman filter paper (No.1). The filtrate was dried at a temperature of 30 °C for 10 hours to produce a gel-like extract, which weighed 20.5 g. Appropriate concentration of the extract was then subsequently made by dilution with distilled water into 200 mg/kg body weight and administered to the animals.

### Handling and treatment of animals

A total of 24 adult albino rats (12 males and 12 females) weighing between 150 – 300 g obtained from the disease free stock of the animal house, Biochemistry Department, College of Medical Sciences University of Calabar, Calabar Nigeria, were used for the study. The rats were divided according to sex into six groups with six rats each, as follows: Group Mc (Male control group receiving distilled water as placebo); Group Fc (Female control group receiving distilled water as placebo); Group Mt (Male test group receiving aqueous extract of P. guajava leaves); Group Ft (Female test group receiving aqueous extract of P. guajava leaves).

The rats were acclimatized in the experimental animal house for one week before the commencement of the experiment. The animals, housed in stainless steel cages under standard conditions (ambient temperature, 28.0 ± 2.0 °C, and humidity 46%, with a 12 hr light/dark cycle), were fed with the normal rat pellets. All the rats in both test and control groups were allowed free access to food and water ad libitum, throughout the experimental period. Good hygiene was maintained by constant cleaning and removal of feces and spilled feed from cages daily.

The animals were administered various agents as follows, Mt and Ft groups received 200 mg/kg body weight oral daily doses of aqueous extract of P. guajava leaves using orogastric tubes and syringes. This lasted for a period of 30 days and the experiments were conducted between the hours of 09.00 am and 10.00 am daily. Rats in the control groups, Mc and Fc, were administered, by oral gavage, with 5 ml of distilled water (placebo). All the animal experiments were carried out in accordance with the guidelines of the Institution’s Animal Ethical Committee.

### Collection and analyses of blood

All the animals were anaesthetized with chloroform vapor, twenty-four hours after last day of extract administration, and dissected for blood collection. Blood samples were collected by cardiac puncture into two sets of plain and EDTA-treated sample bottles, respectively. The blood in the plain sample bottles were allowed to clot, after 3 hours. The clotted blood samples were spun in a bench top centrifuge (MSE, England) to obtain sera. The serum samples were separated into another set of plain sample tubes. The separated serum samples were stored in the refrigerator until required for the enzyme analyses. The whole blood collected into EDTA-treated sample bottles were used for hematological indices. All assays were done within 24 hours of the sample collection. The serum enzymes, alanine amino transferase (ALT), aspartate amino transferase (AST), alkaline phosphatase (ALP), total proteins and albumins assays were carried out according to the procedures described by Randox Laboratories Ltd, United Kingdom. The liver tissues were subjected to normal routine histological procedures, stained with Hematoxylin-Eosin and examined using the light microscope for any morphological changes.

The hematocrit or packed cell volume was determined according to the hematocrit method, while the hemoglobin concentrations were determined by cyanomethaemoglobin method, described by Alexander and Griffiths [23]. The red blood cells count was estimated by the visual method described by Dacie and Lewis [24].

### Statistical analysis

The results obtained from this study were analyzed by one-way analysis of variance (ANOVA), followed by Student’s t-test to evaluate the significance of the difference between the mean value of the measured parameters in the respective test and control groups. A significant change was considered acceptable at P < 0.05. Results of the biochemical estimations are reported as Mean ± SD. Statistical analysis was performed using student’s t-test and P ≤ 0.05 being considered statistically significant.

## Results

**Figure 1:**
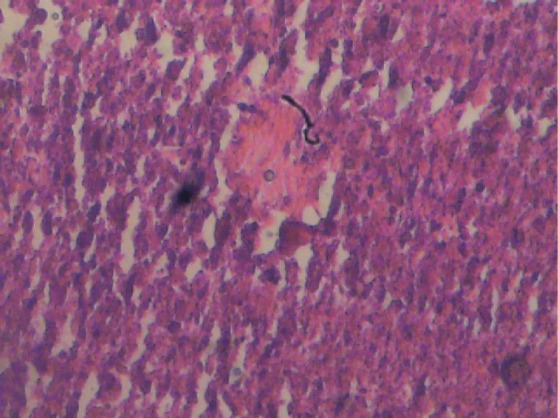
Liver section of male control rat. Cords of hepatocytes well preserved and essentially normal, cytoplasm not vacuolated, sinusoids well demarcated, no area of necrosis, no fatty degeneration and change. Haematoxylin and Eosin stained.

**Figure 2:**
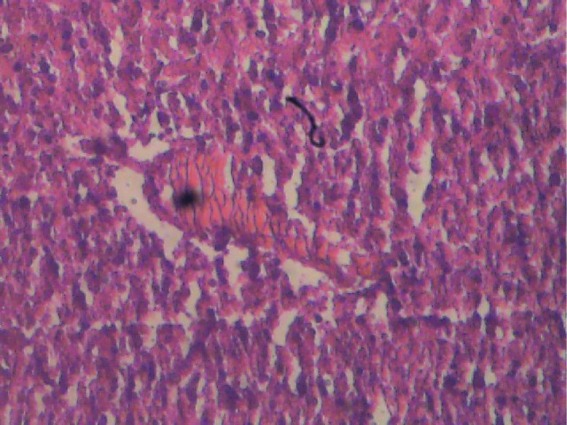
Liver section of female test rat, treated with aqueous extract of P. guajava leaves. Cords of hepatocytes are distinct and essentially normal, no fatty change, cytoplasm not vacuolated. Haematoxylin and Eosin stained.

**Figure 3:**
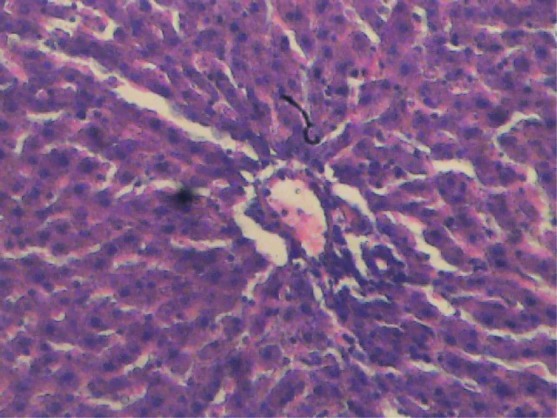
Liver section of female control rat. Cords of hepatocytes well preserved and essentially normal, cytoplasm not vacuolated, sinusoids well demarcated, no area of necrosis, no fatty degeneration and change. Haematoxylin and Eosin stained.

**Figure 4:**
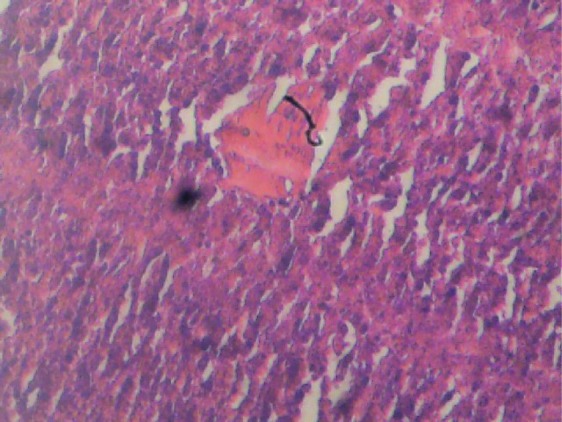
Liver section of female test rat, treated with aqueous extract of P. guajava leaves. Cords of hepatocytes are distinct and relatively normal, no fatty change, cytoplasm not vacuolated. Haematoxylin and Eosin stained.

The results of this study are shown in Tables 1 - 3, as well as Figure 1 - 4. In this study, the preliminary phytochemical screening of the leaves indicated the presence of alkaloids, flavonoids, glycosides, polyphenols, reducing compounds, saponins and tannins (Table 1). However, the proportion of tannins detected to be present in the leaf extract was relatively lower than the other phytochemicals. The results obtained in this present study for some serum liver diagnostic enzymes, total proteins and albumin in normal male and female rats treated with aqueous extract of P. guajava leaves are presented in Table 2, while the results of hematological analysis are presented in Table 3. It was observed from these results that treatment of rats with aqueous extract of P. guajava leaves have no significant effect (P ≥ 0.05) on the activities of the serum liver diagnostic enzymes as well as the concentrations of serum total proteins and albumins, compared respectively with the control (Table 2). This observation indicated that the aqueous extract of P. guajava leaves did not exhibit any remarkable hepatotoxic effect in the animal model.

**Table 1:** Phytochemical Profile of Aqueous Extract of P. Guajava Leaves

Phytochemical Components	P. Guajava Leaf Extract
Alkaloids	++
Flavonoids	++
Glycosides	+
Polyphenols	++
Reducing Compuonds	++
Saponins	++
Tannins	+

++ = Highly present, + = Present

**Table 2:** Effect of P. Guajava Aqueous Leaf Extract on Serum Enzymes, Total Proteins and Albumin Levels of Normal Wistar Rats

Group	ALT (IU/L)	AST (IU/L)	ALP (IU/L)	Total Serum Protein (g/l)	Serum Albumin (g/l)
Mc	9.02 ± 2.10	7.12 ± 2.18	94.20 ± 4.82	60.10 ± 5.00	26.02 ± 2.67
Mt	8.86 ± 2.00*	6.94 ± 1.86*	93.72 ± 5.06*	58.98 ± 6.85*	25.86 ± 2.58*
Fc	9.00 ± 1.95	6.86 ± 2.00	93.85 ± 6.02	59.65 ± 4.89	25.98 ± 2.63
Ft	8.97 ± 2.16*	7.03 ± 1.56*	94.12 ± 5.62*	60.08 ± 5.24*	26.14 ± 2.52*

Values are presented as mean ± Standard Deviation, n = 6, *P ≥ 0.05 compared with the control. Mc = Male control; Mt = Male test,treated with the extract; Fc = Female control; Ft = Female test, treated with the extract.

**Table 3:** Effect of Ascorbic Acid and Aqueous Extract of P. Guajava Leaves on Some Haematological Indices in Male Rats

Group	PCV (%)	Hb (g/dl)	RBC (x10^6^ cells mm^-3^)	WBC (x10^3^ cells mm^-3^)
Mc	41.36 ± 2.23	12.10 ± 2.50	1.30 ± 0.05	4.50 ± 2.20
Mt	47.80 ± 1.88*	16.02 ± 1.86*	1.58 ± 0.06*	4.53 ± 2.11**
Fc	40.86 ± 2.00	11.68 ± 2.00	1.29 ± 0.07	4.48 ± 1.96
Ft	47.02 ± 2.55*	15.86 ± 1.98*	1.56 ± 0.06*	4.50 ± 2.00**

The data are presented as mean ± SD, n = 6. *P < 0.05 compared with control, **P ≥0.05 compared with control. Mc = Male control; Mt = Male test,treated with the extract; Fc = Female control; Ft = Female test, treated with the extract

Figures 1 and 2 show histological sections of the liver tissues of the control and test male rats, while figures 3 and 4 show the histological sections of the liver tissues of the control and test female rats, respectively. The liver section of the animal in control groups showed a central vein with prominent small-sized nuclei, with the hepatocytes well separated by sinusoids. While the tissue section of the test rats showed a prominent central vein with a relatively large-sized nuclei; also, the sinusoids separating the hepatocytes in the test rats are observed to be relatively more prominent than that of the rats in the control groups. Generally, the liver sections of both male and female rats in the control and test groups showed that the cords of hepatocytes well preserved, cytoplasm not vacuolated and the sinusoids well demarcated. Also, no area of infiltration by inflammatory cells and fatty degenerative changes were observed in the tissue sections. These features gave an indication of normal hepatic architectural integrity for male and female rats in both control and test groups.

The results of this study also showed that the red blood cell counts, hematocrit and hemoglobin concentrations increased significantly (P < 0.05) on administration of the extract to both male and female rats. Also, no significant effect (P > 0.05) on the white blood cell counts was observed to be associated with treatment of both male and female rats with extract (Table 3).

The observations made from these results also indicated that the hematopoietic effect of the extract on male rats was not significantly different (P > 0.05) from the effect on the female rats. This present study therefore suggests that aqueous extract of psidium guajava leaves is not hepatotoxic, but rather possess hematopoietic property in both male and female rats. These findings are therefore of clinical importance given the various reported medicinal potentials of the plant.

## Discussion

Plants generally have varied chemical compositions (referred to as phytochemicals) depending upon species. A good number of plants are known to be of economic and medicinal value. Those that are of medicinal value are often used as herbal remedy for the restoration and maintenance of good health. Some herbs have been considered as drugs and therefore generally safe and effective [25]. Most herbs have been associated with broad actions on a number of physiological systems in concert unlike the pharmaceutical drugs which are usually designed to elicit a specific effect. Some researchers on medicinal plants are of the opinion that some herbal plants are usually oriented in the same general therapeutic direction and are complementary or synergistic, often non-specific but very rarely adverse [25, 26]. The potential of the leaf extract of P. guajava may be related to its antioxidant activity. In our routine laboratory studies, we observed that aqueous extract of P. guajava leaves exhibited the same effect on lipid profile as ascorbic acid in rats. The extract contains flavonoids which are powerful antioxidant polyphenolic compounds. Torell et al [27] and Faure et al [28] have shown that flavonoids inhibit peroxidation of polyunsaturated fatty acids in cell membranes. Moreover, reports have shown that flavonoids from Helichrysum genus inhibit the formation of superoxide ions and hydroxy radicals, which are two strong peroxidation agents [29].

From the results of this investigation, hepatocellular function-enhancing effect of the aqueous extract of P. guajava leaves is reported. Generally, analyses of the activities of some basic liver function enzymes in the plasma or serum can be used to indirectly access the integrity of tissues after being exposed to certain pharmacological agent(s). These enzymes are usually liver markers whose plasma concentrations above the homeostatic limits could be associated with various forms of disorders which affect the functional integrity of the liver tissues. Preliminary phytochemical screening carried out in this study indicated that P. guajava leaves contain alkaloids, flavonoids, tannins and saponins in its aqueous extracts. These phytochemicals are known to perform several general and specific functions in plants, and may exhibit different biochemical and pharmacological actions in different species of animals when ingested. These actions range from cell toxicity to cell protective effects [11]. The phytochemical report of this study suggested that the hepatocellular function-enhancing effect of the extract may result from the action of the various extracts’ contents, especially the presence of flavonoids which have been reported to have antioxidative effects [30]. Furthermore, saponins also present in the extract are known to have hypocholesterolemic activities [31], which may aid in lessening the metabolic burden on the liver. In this study also, a non-significant effect of the aqueous extract of P. guajava leaves on the liver function enzymes and the morphological architecture of the liver tissues is reported. The results of this present study therefore showed that the use of the aqueous extract of P. guajava leaves as liver tonic in some part of the world may not interfere with the functional integrity of the liver tissues.

The results of this study also indicated that the extract of P. guajava leaves may possibly serve as an acceptable blood booster in an anemic condition or prophylactic purpose. Although the specific mechanism(s) through which the extract facilitated the increase in these hematological indices was not ascertained in this study, this action is assumed to be a direct effect of the extract on the haematopoietic systems. It is possible that the extract contains such constituent(s) that can interact and stimulate the formation and secretion of erythropoietin, hematopoietic growth factors/committed stem cells. Specifically, stimulations of hematopoietic growth factors and erythropoietin systems have been reported to enhance rapid synthesis of blood cells [32]. Moreover, the hematopoietic potential of the leaf extract of P.guajava may be related to its antioxidant activity. The phytochemical screening showed that the extract contains flavonoids which are powerful antioxidant polyphenolic compounds. Torell et al [27] and Faure et al [28] have shown that flavonoids inhibit peroxidation of polyunsaturated fatty acids in cell membranes. Also, flavonoids from Helichrysum genus have been reported to inhibit the formation of superoxide ions and hydroxy radicals, which are two strong peroxidation agents [29]. This antioxidant activity may protect both the hematopoietic committed stem and the formed blood cells from the attack of the reactive free radicals in the body.

In conclusion, the results of the present study indicated that aqueous extract of P. guajava leaves possess hematopoietic potential without any significant hepatotoxic effects in rats. Hence, the aqueous extract of plant’s leaves may be suggested to possess hepatoprotective potency.
